# Children Learning About Second-hand Smoke (CLASS II): a mixed methods process evaluation of a school-based intervention

**DOI:** 10.1186/s40814-021-00853-9

**Published:** 2021-05-24

**Authors:** Cath Jackson, Rumana Huque, Farid Ahmed, Shammi Nasreen, Sarwat Shah, Jasjit S. Ahluwalia, Mona Kanaan, Aziz Sheikh, Kamran Siddiqi

**Affiliations:** 1 Department of Health Sciences, University of York, ARRC Building, Heslington, York, Y010 5DD UK; 2 Department of Economics, University of Dhaka and ARK Foundation, House No 6, Road NO 109, Gulshan 2, Dhaka, Bangladesh; 3 ARK Foundation, House No 6, Road NO 109, Gulshan 2, Dhaka, Bangladesh; 4 Department of Behavioral and Social Sciences, Brown University School of Public Health, 121 South Main St, Providence, RI 02912 USA; 5 Usher Institute of Population Health Sciences, The University of Edinburgh, Edinburgh, EH8 9DX UK

**Keywords:** Second-hand smoke, Smoke-free homes, Process evaluation, School

## Abstract

**Background:**

Children are vulnerable to the effects of second-hand smoke exposure. Creating smoke-free homes is an effective strategy to limit exposure. We developed a smoke-free intervention (SFI) using children as a catalyst for change and teaching skills to negotiate a smoke-free home. In this paper, we present the process evaluation conducted within a pilot trial.

**Methods:**

This was a mixed-methods study comprising qualitative interviews and quantitative fidelity assessment of SFI delivery. Interviews in the six intervention schools were conducted with six headteachers and 12 teachers. These explored experiences of delivering the SFI, perceived impact, barriers and facilitators to success, and ideas for improvement and for scaling up. The data were analysed using framework analysis. Delivery of the SFI was observed and fidelity scores calculated.

**Results:**

The SFI was acceptable to headteachers and teachers. Fidelity scores ranged from 27/40 to 37/40. Didactic components were more fully implemented than interactive components. Time to complete the sessions, timing in the school day and school calendar were key challenges. Embedding the SFI into the curriculum was a potential solution.

**Conclusions:**

These findings provide useful information to finalise the content and delivery and inform the scale-up of the SFI for our definitive trial, which is now underway.

**Trial registration:**

ISRCTN68690577

## Key messages regarding feasibility

- We wanted to explore the implementation, mechanisms of impact, and contextual influences for our smoke-free intervention (SFI)
- The SFI was delivered with enthusiasm, with didactic components being more fully implemented than interactive components; time and timing of the sessions were a challenge within the busy school schedule and curriculum.
- More training on engaging students in active discussion is needed, and early engagement with schools to plan delivery of the SFI.

## Background

In 2016, second-hand smoke (SHS) exposure caused an estimated 884,000 deaths and a loss of 24 million disability adjusted life years worldwide [1]. Almost a third of this disease burden was among children. Considered particularly vulnerable to SHS exposure, children are at risk of sudden infant death syndrome, meningitis, respiratory and middle ear infections, asthma and asthma exacerbations [2–4]. In many countries, the majority of children are exposed to SHS; for example, in a 2019 school-based survey in Dhaka, Bangladesh, cotinine was detected in the saliva of 95% children aged 9–11 years old—a possible indicator of SHS exposure [5]. Homes are often the primary source of children’s exposure to SHS. A recent study of 1746 households in Mirpur, Dhaka found that smoking inside the home was permitted in over half (55%) of households [6]. After smoking bans in public and workplaces, creating smoke-free homes (SFH) is an effective strategy to limit exposure to SHS [7].

Schools are an important health promotion setting, providing an opportunity for delivering health promotion education as well as creating healthy school environments [8]. School-based interventions targeting smoking typically focus on preventing initiation of smoking or reducing students’ smoking behaviour, and evidence of effectiveness is mixed [9–12]. Schools can also harness the potential of children as a catalyst for change [8, 13] and teach skills to negotiate a SFH to reduce tobacco exposure. To date, only a handful of studies have explored this approach [13, 14], typically as part of a wider intervention. In a Chinese study, Wang et al. [15] reported a reduction in children’s SHS exposure at 6 months following an intervention combining health education with children and cessation support for their parents. In an older Chinese study [16], one component of a school-based intervention encouraged school children to write a letter to their smoking fathers asking them to quit. Students reported a decrease in the fathers’ smoking rate. Finally, in Bangladesh, Huque et al. [17] assessed the feasibility of a smoke-free intervention (SFI) that is the focus of this paper. They concluded it had potential to encourage children to negotiate a SFH.

SFI is a theory-based behaviour change intervention developed by a multidisciplinary group in Bangladesh and the UK [17–19]. It is delivered to year 5 children (aged 9–11 years) by teachers who are provided with training and resources. It consists of two 45-min sessions delivered over two consecutive days (see Table 1). Session 1 focuses on delivering a classroom presentation with discussion (flipchart activity). Session 2 involves storytelling with role play, quiz, and word search. The presentation, quiz, and games aim to make children aware of the harms of SHS and motivate them to achieve a SFH. The storytelling and role-play activities focus on building children’s confidence in raising their concerns about SHS with their parents and enhance their negotiation skills. Four refresher sessions (15 min each) follow over the subsequent 4 weeks. These reinforce learning by revising salient points of the initial sessions and by encouraging children to share their experiences of initiating conversations with their families. Teachers also help children to plan their next action and overcome any challenges they face. Children are provided with take-home promise forms for families that provide graphic representations of the hazards of SHS, pictorial guidance to help them create a SFH, and a tear-off slip to commit to imposing smoking restrictions at home. Teachers are also trained to pick up any signs of distress among children as an untoward consequence of SFI.

**Table 1:** Smoke-Free Intervention topics, components, and corresponding fidelity scores

	School 01-01	School 01-03	School 01-06	School 02-03	School 02-04	School 02-06
**Session 1—flipchart activity**						
**A. Explain about second-hand smoke (SHS) and imagine a smoke-free home (SFH)**						
Introduce the story characters	2	2	2	2	2	2
Smoky family picture	1	2	2	2	2	1
Transformation from smoky home to SFH	1	2	2	1	2	2
Describe a SFH	2	2	2	2	2	2
**B. Highlight the adverse events of SHS**						
No-smoking advert on TV	1	2	2	2	2	2
The respiratory effect of SHS	1	2	2	1	2	2
Effects of SHS on heart and Lungs	2	2	2	2	2	2
**C. Explain about the chemicals in tobacco smoke and diseases associated with smoking**						
Chemicals in tobacco smoke	2	2	2	2	2	2
Smoking can cause many diseases	1	2	2	2	1	2
**D. Teach negotiation skills**						
Ideas about negotiation with smoker	1	2	1	2	1	1
Avoiding exposure to SHS	1	2	2	0	1	1
Difficulty of changing smoker’s behaviour	1	1	1	2	1	0
Bijoy negotiating with his Father	1	2	2	1	2	1
Negotiating with visitors	1	2	2	2	1	1
**E. Plans to create a SFH**						
Making the home completely smoke free	1	2	1	1	1	2
Explain self-monitoring	1	1	1	2	2	1
Encourage students to follow the steps to make their homes smoke free	1	1	1	2	1	1
**Session 2**						
**F. Class activities**						
Storytelling with role play	2	2	2	2	2	2
Word search	2	2	2	2	2	2
Quiz	2	2	2	2	2	2
**Total score for school**	**27**	**37**	**35**	**34**	**33**	**31**

*Note.* 0 = not implemented, 1 = partially implemented, 2 = fully implemented. Maximum possible score is 40.

Following a successful feasibility study (CLASS I, Children Learning about Second-hand Smoke [17], we conducted a pilot cluster randomised controlled trial (CLASS II) in 12 schools in Bangladesh [20, 21]. The aims were to (1) seek preliminary evidence of effectiveness; (2) test the methods for recruitment, randomisation, and collection of outcome measures; and (3) explore the implementation, mechanisms of impact and contextual influences for the SFI. The findings for aims (1) and (2) are summarised in Table 2 and reported elsewhere [21]. This article addresses aim (3) and presents the findings of process evaluation that was embedded within the pilot trial.

## Methods

This was a mixed-methods study comprising qualitative interviews and quantitative fidelity assessment of the SFI delivery [20].

### Interviews

#### Participants and setting

Of the six co-educational schools that received the SFI intervention, three were government and three private, located in Mirpur (urban) and Savar (peri-urban), Dhaka. Participants were recruited from all six intervention schools and included the six headteachers (all male) and the 12 teachers (10 male, 2 female) who delivered the SFI (see Table 3 for school and participant characteristics).

**Table 2:** Feasibility and preliminary effectiveness findings for CLASS II

Twelve schools were recruited. Six were randomly allocated to the smoke-free intervention (intervention arm, n = 245 children), and six delivered usual education only (control arm, n = 236 children). Of 481 children who had cotinine levels indicative of second-hand smoke exposure, 450 were followed up (229 intervention arm; 221 control arm). All schools were retained in the study; 89.9% children (206/229) in the intervention arm and 86.8% (192/221) in the control arm provided a saliva sample for cotinine 2 months post-allocation. Mean cotinine at the cluster level was 0**.**53 ng/ml (standard deviation 0.36) in the intervention arm compared with 1**.**84 ng/ml (standard deviation 1**.**49) in the control, a mean difference of – 1**.**31 ng/ml (95% confidence interval − 2**.**86, − 0**.**24). In summary, it was feasible to recruit, randomise, and retain primary schools and children. This study, though not powered to detect differences in mean cotinine between the two arms, provided estimates to inform the likely effect size for a future trial.	

The six headteachers had 16–33 years’ teaching experience. All but one had experience of implementing health projects in their school, in the areas of handwashing, clean-water management, disaster management, health education, family problems, and general hygiene.

Each headteacher identified the teachers who had delivered the SFI to be interviewed (two per school). Teachers’ experience ranged from 3–21 years. They had experience of delivering health projects in the areas of handwashing, clean-water management, nutrition, learning disabilities, and child safety.

#### Data collection

Semi-structured interviews occurred October 2015–July 2016, after the SFI had been delivered in each school. The local research team (SN, FA, MG) conducted the interviews face-to-face, on school premises. A topic guide was used to ensure consistency across interviews. The format was flexible to allow participants to voice what they considered to be important. The interviews were digitally audio recorded. All participants received study information and provided written informed consent before the interview commenced.

Interviews with headteachers explored their views on the implementation of the SFI within their school including its content, perceived impact, barriers and facilitators to success, the enthusiasm of their staff, usefulness of the training and manual, potential/challenges of scaling up and of working in partnership with non-government organisations on projects of this type.

Interviews with the teachers explored their views and experiences of delivering the SFI, asking in detail about implementation of the different components, what had gone well and why, any challenges, ideas for improvement and perceived impact. The interview concluded with a reflection on the training session and manual, and a discussion of how the intervention could be continued and scaled-up.

Interviews with headteachers lasted 30–40 min. Those with the teachers lasted 40–50 min.

#### Data analysis

The interviews were transcribed verbatim and translated into English by FA and SN. The data were subjected to thematic analysis using the Framework approach [22] which is designed to address policy and programme-related questions. The data analysis team was composed of three local researchers (RH, FA, SN) and a senior qualitative researcher in the UK (CJ). QSR NVivo 10 [23] software package facilitated data management. The following steps were undertaken:

Familiarisation: The data analysis team read the first three teacher interview transcripts and one headteacher transcript to record emerging ideas and recurrent themes that were relevant to the study aims.

Constructing a thematic framework: A thematic framework was developed by FA and TA. It was structured by the topic guide and ideas and themes from the previous step. CJ independently reviewed the framework and refined it where necessary. The same framework was used for the teacher and headteacher interviews, except that four additional themes (teachers’ participation in SFI, scaling-up, extending SFI to other schools, partnership) were included in the framework to capture the interview data from headteachers.

Indexing and charting: The thematic framework was then systematically applied to the interview data by FA and TA. Charts were produced in NVivo for each theme, and summaries of responses from participants and verbatim quotes were entered. CJ reviewed a random sample of 20% of the completed charts to check the accuracy of the summaries and quotes.

Mapping and interpretation: The completed charts were reviewed and interrogated by FA and TA to compare and contrast views, seek patterns, connections, and explanations within the data. Negative cases (with opposing views to the majority) were actively sought. Descriptive findings documents were written for each theme. CJ reviewed each document to check that the interview data were captured appropriately and then mapped the themes to the UK Medical Research Council’s process evaluation functions [24], identified as:

- Implementation—How is delivery achieved, and what is actually delivered?
- Context—How do factors external to the intervention affect implementation and outcomes?
- Mechanisms of impact—How does the delivered intervention produce change?

### Fidelity assessment

#### Data collection

Drawing on guidance for best practice for fidelity assessment [25], delivery of the two 45-min sessions in each intervention school were observed by a member of the local research team (FA) who completed a 20-item fidelity index. The index was developed by the team who created the SFI, which included behavioural scientists. Each item corresponded with the 20 components of the SFI, that were grouped into six topics A to F (see Table 1). Delivery of each component was scored as 0 (not implemented), 1 (partially implemented), or 2 (fully implemented). Definitions were provided for each component (available from authors on request); for example, “effects of SHS on heart and lungs” was scored as:

- 0 = Skipped the slide
- 1 = Delivered component (a) talked about the lead character’s (called Bijoy) football coach and described the importance of having a healthy heart and lungs to be a happy football player OR component (b) introduced the concept of SHS and its harms to heart and lungs by prompting active learning through questions.
- 2 = Delivered both components (a) and (b)

#### Data analysis

For each school, we computed a total fidelity score by summing the scores for all 20 items (SFI components) in the fidelity index, providing a range of 0 (did not implement any SFI components) to 40 (all SFI components were fully implemented). For each topic, we counted the number of times each component was fully implemented and divided this by the total number of opportunities for full implementation; for example, for topic E “plans to create a SFH”, total number is 18 (6 schools × 3 components). These data were then triangulated with the relevant interview data about implementation.

## Findings

The findings are organised by the three functions for process evaluations: implementation, context, and mechanisms of impact [26]. Where headteachers and teachers offered views, both perspectives are presented. If only teachers or headteachers spoke of an issue, this is evident because only those accounts are included.

### Implementation: how is delivery achieved (training and resources)

All teachers had received 1 day of training on the SFI, delivered in August 2015, once in Mirpur (hosted in a local NGO office) and once in Savar (hosted by a school). The training comprised an overview of the CLASS II trial protocol, a quiz about SHS and SFH followed by a presentation on these topics, discussion of the SFI schedule and role of the teacher, followed by participation in the intervention components: flipchart, story book, role play, word search, and quiz. Teachers were provided with an intervention manual and intervention resources to take away.

**Table 3:** Characteristics of the intervention schools, teachers, and headteachers

	Total	ID 01-01	ID 01-03	ID 01-06	ID 02-03	ID 02-04	ID 02-06
Type of school	-	Private	Public	Public	Private	Private	Private
Location	-	Urban	Urban	Urban	Peri-urban	Peri-urban	Peri-urban
	N	N	N	N	N	N	N
Teachers							
Male	10	1	2	2	2	2	1
Female	2	1	0	0	0	0	1
Years’ teaching experience							
Less than 5 years	3	1	0	0	2	0	0
5-10 years	5	0	0	1	0	2	2
10 years +	4	1	2	1	0	0	0
Headteachers							
Male	6	1	1	1	1	1	1
Female	0	0	0	0	0	0	0
Years’ teaching experience							
Less than 10 years	1	0	0	1	0	0	0
10 years +	3	0	1	0	1	1	0
Not reported	2	1	0	0	0	0	1

Overall, this training was well received. Some teachers said that they had already known about SHS, but the training had provided them with more facts as well as with ideas on how to share this knowledge with the children, their own families and friends. For others, the training provided them with new knowledge about SHS and achieving a SFH (Table 4, quote 1). Two teachers offered thoughts on the most useful component of the training, namely the opportunity to share and discuss their ideas for delivering the SFI with other teachers and the written materials on SHS.

**Table 4:** Illustrative quotes—implementation, context, and mechanism of impact

Quote ID	Quote and participant ID
**Implementation**	
1	*Of course it (the training) was helpful. I didn’t have much knowledge about SHS earlier. But after this project I got to know about it at a larger scale. Then I learnt how harmful smoking is for our family and surroundings, how to keep ourselves away from it, and how we can motivate our children to keep our homes smoke-free.* 02-05 Teacher
2	*Besides, you can train the team leaders or student girl guides and prepare them as our co-warrior. You can nominate 2 persons from each class. The way they can reach to their classmates, we may not. One friend can discourage another friend while smoking, but the same student may not smoke in front of his teachers at the first place.* 01-01 Teacher
3	*As far as I understood from the story, Bijoy is a restless young boy. I kept that in mind while choosing this character from my students. Bijoy’s sister was expert with words, that’s why I chose one of my students who sings and presents well. That’s how I chose other characters, too. So that they can play their role perfectly.* 02-06 Teacher
4	*How well the children did, what they did with the promise forms and then, the way we guided them to keep their home smoke free, which day their home was smoke free or which day it wasn’t. Then we asked them if they faced any problem at home. I also asked them how their fathers smoke now, if they still smoke in front of their kids, do they smoke outside of the homes, or in a closed room.* 01-05 Teacher
5	*We could explain the necessary information on SHS through photos and story. I think it was very effective way of giving children the confidence of talking with their parents and making their home smoke free.* 01-02 Teacher
6	*Moreover, when children collected signatures on the promise forms from the parents, their parents realised that children are now aware of the issues. So they should feel the necessity to stop smoking in front of them. It is not easy to quit smoking. But the parents who put signatures on the promise forms at least tried to follow the advice.* 02-05 Teacher
**Context**	
7	*Not all the parents are sincere about such issue if this is included in their children’s curriculum, they will get to know about it too they will feel the incentive that children should know about it and if this is in the curriculum, it will have marks allocated so parents will be sincere about it.* 01-05 Teacher
8	*No, it should better stay as a separate programme, because this is curriculum made by the Government. The Government always discourage smoking in different ways through stories, plays. This programme does not need to be included in the textbooks…if it could be arranged anytime other than class hours that would be better.* 02-03 Headteacher
9	*The bad effects of smoking are discussed briefly in the science textbook. For example, diseases that are caused from smoking these are discussed in a chapter called infectious disease. How harmful smoking inside a closed-door room is, what is second hand smoking in brief. But your project discussed about this issue in details. And your project can make a very good chapter for textbook.* 02-05 Teacher
10	*For a good partnership, if you can consider and maintain the timing of the school and classes of the children so their normal education is not hampered in anyway, then any good work can be accomplished through partnership.* 02-03 Headteacher
**Mechanisms of impact**	
11	*Children liked the flipchart containing photos, and the role play. They could learn practically from that. They gained insight on what to do in such situations. They realised the overall issue*. 02-06 Teacher
12	*Reading out the story or doing acting on it means they will keep it in their mind for longer period of time. And students could understand the meaning of the story because of characters like Bijoy and Bithi. So the role-play and the storybook, both were very helpful for the students to grasp the main points of your project.* 02-03 Teacher
13	*Second hand smoking is an unavoidable social problem; we should get aware of this problem immediately. I think, children are the key motivator in a family, so if we teach the children on how to make their home smoke free, they will try to follow it at their home. When they will get aware of second hand smoke they will be able to negotiate with their parents to stop smoking at home.* 01-04 Teacher
14	*Earlier, I didn’t know that second-hand smoking is this much injurious to children. I didn’t have much knowledge about it and the diseases it causes. I thought only smokers get affected from smoking. Now when someone smokes near me, I feel like I am being affected by it and nicotine is entering into my body, too. Therefore, I must say that I have taught a lot from your project the diseases that smoking can cause etc. I still have the book you gave me, and I often read it.* 02-03 Teacher

A few suggestions were offered to improve the training: first, to provide it to all teachers to develop capacity for delivering the programme within the school. The second idea was to deliver the training over 2 or 3 days to have more time to cover the material and for “hands on” training. It also was suggested to have a refresher session every few months to maintain the teachers’ enthusiasm, remind the children not to smoke themselves, and to monitor the impact of the SFI. Finally, a teacher and a headteacher (from the same school) suggested training two children from each class to be “co-warriors” who would support their classmates with the SFI (Table 4, quote 2).

### Implementation: what was delivered (quantity)

Four schools delivered all 20 SFI components (see Table 1). Two schools each missed delivering one component of negotiation skills (topic D, avoiding exposure to SHS, difficulty of changing smoker’s behaviours).

Teachers described delivering the SFI to between 40 and 60 children. Approximately half had run the two sessions after the timetabled classes had ended. Others had run the SFI sessions instead of timetabled classes, specifically mathematics or science. One headteacher was willing to cancel scheduled classes to deliver the SFI because the school was committed to this type of project.

The length of the two sessions (designed to be 45 min) ranged from 40 to 100 min. Most teachers commented that 45 min were insufficient and that between 5 and 60 additional minutes were needed to complete activities and encourage discussion, particularly in larger classes. A few stated that 45 min was acceptable if the session was well planned; any longer, the children would get bored.

The four refresher sessions had been delivered at a variety of times in the school day: in the first class or last class of the day, after finishing class tests, or after class. Teachers reported that these sessions typically lasted 10–25 min. Half said that 15 min was sufficient, whilst others needed five more minutes.

In discussing the intervention components within the two SFI sessions (1. flipchart; 2. Storytelling with role play, word search, and quiz), the teachers explained how they had tailored these to best suit their class. In selecting children for the role play, most teachers had asked for volunteers; however, a few had selected children either because they were perceived to be like the characters in the story or because they were confident performers (Table 4, quote 3). Three teachers described how some children, particularly girls, were shy at first about the prospect of acting. For the storytelling, some teachers had read the story to the children, some asked for volunteers or selected children to read to the class, whilst others gave the book for the children to read themselves. Finally, two methods of organising the quiz activity were evident; several teachers read out the questions to the whole class, and the children raised their hands to answer; others divided the class to compete as quiz teams.

A range of activities had been covered within the refresher sessions: collecting in the promise forms, reminding children of what they had learnt about SHS, asking them to share what was happening at home with their father’s smoking, and providing further encouragement on negotiating with parents (Table 4, quote 4).

### Implementation: what is delivered (quality)

The fidelity scores across the six schools ranged from 27 to 37 of a maximum possible score of 40 (see Table 1). The components that were less well delivered, across all schools, related to topics D and E: teaching negotiation skills (37% full, 57% partial, 6% no implementation) and plans to create a SFH (28% full, 72% partial implementation). The didactic topics A to C (explaining about SHS and imagining a SFH, highlighting the adverse events of SHS, explaining the chemicals and diseases) were better delivered (83% full, 17% partial implementation for each topic). Notably, all components (topic F) on day 2 (storybook, word search, quiz, promise forms) were fully (100%) implemented.

Consistent with the fidelity data, teachers were unanimously positive in their overall assessment of the SFI delivery. They were asked to reflect on how well the different components had gone, and to propose improvements to their content and delivery. They all liked the pictures in the flipchart which they believed had held the children’s attention, helped them to understand the messages, and to develop confidence to speak with their parents about having a SFH (Table 4, quote 5). To improve the activity, two teachers and three headteachers suggested replacing the flipchart with a more modern multimedia presentation.

The majority view was that the other components in the two sessions did not need improvement, as they were informative, and the children had enjoyed them, as evidenced by their enthusiastic participation. Some teachers suggested including more harmful effects of SHS within the role play and storytelling, as well as adding more words into the word search (perhaps in English) and more questions into the quiz. These ideas were all seen to provide children with even more information about SHS.

The general perception was that the children had understood the purpose of the promise form and were very motivated to take it home. One teacher had encouraged the children to talk to their parents about the story when discussing the promise form. Most teachers said that the children had brought back the promise forms signed by their fathers and had reported no problems in discussing it with their parents. Some described the children’s accounts of using the promise form: some fathers had been reluctant at first to put up the SFH signs then did so later; parents had learnt of the risks of SHS through conversation with their child, and some parents had tried to create a SFH (Table 4, quote 6). There were no suggestions on how to improve the promise form.

### Context: factors external to the intervention which affect implementation and outcomes

An important contextual factor affecting delivery of the SFI was timing within the school year. One teacher suggested that the SFI should be delivered to other year classes or scheduled in December to avoid exams. Four teachers spoke of the difficulty for the children to find time to complete the symptom diary (not part of SFI, instead a data collection tool for the evaluation) because of their intense studies leading up to Class 5 exams. One said that because of this pressure, many children had lost their diary and stickers; adding that it was difficult to keep track of the children and their diaries once they had moved to Class 6.

Context was seen as particularly important in achieving sustainable delivery of the SFI. Several teachers and two headteachers spoke of the importance of embedding the SFI into the school curriculum so that it becomes compulsory to teach. This was based on a view that it would reach more children, ensure it becomes routine for the teachers and, in studying this topic for exams, would ensure that the children and parents learnt important messages about SFH (Table 4, quote 7). An alternative perspective was offered by one headteacher—namely, that the SFI should not be included in the curriculum and should stay as a stand-alone programme delivered outside of class time. This was because there was already sufficient teaching on smoking in the curriculum, and additional sessions distracted the children from their regular study (Table 4, quote 8).

In discussing where in the curriculum the SFI should be taught, most teachers and headteachers suggested it would sit best in the health-related chapter of the science textbook. Other suggestions were to include it in the social science textbook where the risks of tobacco and addiction were taught, within teaching about the environment, in Islamic studies, as part of listening and reading in English or Bangla lessons, or in a subject recently introduced called ‘Work and Life Oriented Education’. One headteacher declared that where the SFI best fits in the syllabus would depend on whether it was being taught from the perspective of morals or health. A few teachers spoke of the difference of the SFI to other lessons on smoking, particularly that it was longer and more detailed (Table 4, quote 9). For one, this was a barrier to including it within the curriculum.

Finally, a few teachers, and most of the headteachers, observed that the Government of Bangladesh would need to take the decision for the SFI to be included in the curriculum. One headteacher saw that as difficult to achieve because the Government would be concerned about disrupting the schedule of regular classes. Other participants were encouraged that the Government had updated the curriculum to include additional topics, for example HIV, and that a disaster management programme had been integrated into the social science curriculum, due to considerable work by a Non-Governmental Organisation (NGO).

Also viewed, by all headteachers, as important to delivery and sustainability of SFI was schools working in partnership with NGOs. The key facilitators to successful partnership working were seen to be cooperation, not interrupting the everyday running of the school, focusing on benefiting the children, and working together on a regular basis. A few headteachers suggested setting up a team to include 2–3 teachers and a member of the school committee from each local school, to work with NGO representatives (Table 4, quote 10). Half of the headteachers saw no barriers to partnership working. The others identified funding, specifically that NGOs have limited funding and cannot work with every school and that teachers cannot miss classes to attend the aforementioned team meetings.

### Mechanisms of impact: participant responses to, interactions with the intervention

All teachers and headteachers spoke positively about the SFI and its constituent components. The headteachers commented that their teachers had been interested, enthusiastic, and committed to delivering it well even when this had required them to teach extra classes to catch up on routine teaching that was missed due to accommodating the SFI. Participants identified benefits to themselves, the children, and more widely for society. In terms of benefits to themselves, several teachers commented on how much they had enjoyed the experience of delivering the SFI and that it had raised their own awareness of the risks of SHS.

All the teachers and headteachers talked about how much the children had enjoyed taking part in all the SFI activities and that SFI had taught them important messages about the risks of smoking and SHS at an appropriate “*impressionable*” (02-02, Headteacher) age to learn this. The flip chart and role play activities were considered by most teachers to be the activities that the children engaged with most enthusiastically (Table 4, quote 11).

### Mechanisms of impact: mediators

In reflecting on the changes that they observed in the children, teachers all mentioned an increase in their knowledge about SHS and SFH. This was seen to be very important in creating a generation who know the risks of smoking, to tackle the widespread problem of smoking in Bangladesh. They described how children engaged with the messages in the flipchart activity (evidenced by how many questions they asked). It was suggested that storytelling with lots of pictures was a good way to teach the children important SFH messages and to help them to remember these. The pictures with children in them were mentioned as particularly helpful in this, specifically the picture of Bijoy (the boy in the story) and his mother planning together to speak to his father (Table 4, quote 12). The quiz and word search were seen to have taught the children new facts about SHS, for example how much money people spend on smoking, how many people are affected by smoking, how many die from smoking, the risks of SHS, and who is most vulnerable to these risks; as well as new words about SFH and their meanings.

A second change in the children, mentioned by several teachers, was learning negotiation skills and developing confidence in using these skills with their family. This was achieved through the role play activity as well as seeing Bijoy (the boy in the story) successfully achieve a SFH. Two teachers suggested that children had become key agents of change within the family home and so could persuade their parents not to smoke at home, and eventually to not smoke at all (Table 4, quote 13).

### Mechanisms of impact: unanticipated pathways and consequences

Two positive unanticipated consequences of the SFI were reported. One teacher described how he was stopping smokers in the street to try to convince them not to smoke in public places. A headteacher spoke of how the project had resulted in a member of staff no longer smoking on the school premises (Table 4, quote 14).

## Discussion

In this paper, we report the findings of an embedded process evaluation of a theory-based behaviour change intervention (SFI) that targeted school children as an agent of change to achieve SFHs. We used the UK Medical Research Council process evaluation functions (implementation, context, mechanisms of impact) [25] to organise the interview data. This ensured a comprehensive evaluation drawing on recommendations for good practice for process evaluations [25].

Process evaluations provide useful insight into the effectiveness (or not) of complex health interventions including those delivered in schools [8]. This can be particularly helpful where the evidence of their impact is mixed, as it is for school-based health promotion interventions [9–11], because features of success and failure can be identified. In the feasibility/piloting of new interventions, for example the SFI, they offer important information about acceptability to those delivering and receiving the intervention and help refine the content and delivery prior to evaluation.

In terms of acceptability, the SFI, its constituent components, and training were reviewed well by the headteachers and teachers. Teachers reported that their own knowledge had improved, and one had stopped smoking on school premises. Their perception was that the children had enjoyed the activities, learnt important messages, and gained confidence in negotiation skills.

It is recommended that evaluations of intervention fidelity are an integral part of the conduct and evaluation of all health behaviour intervention research [25, 26]. In short, if an intervention is not implemented as directed, and no effect is found, then one cannot be sure whether this is due to lack of efficacy of the intervention or simply that it has not been implemented correctly [25]. A recent example of this is a school-based intervention “Operation Smoke Storm” designed to encourage students to think about the tobacco industry, which was not found to be effective in preventing smoking uptake [27]. The authors offer low self-reported fidelity by teachers as one potential explanation.

The didactic components of the SFI (topics A to C) and the day 2 activities (topic F) were implemented more fully than the interactive components (topics D and E). Feedback from the fidelity assessor suggested that a “partially implemented” score for the interactive components was typically associated with a lack of discussion with the students to elicit their opinions. A key feature of the SFI is student engagement, so where this content was lacking, fidelity was considered to be compromised. Conversely, teachers were encouraged in their training to adapt delivery of intervention components to work best for their students, which they said they did. Hawe et al. [28] endorse this approach, suggesting that by adapting the delivery of component parts of an intervention to the context (in this case, different student classes) can lead to greater fidelity of the intended *function* of the intervention component [26].

Time and timing emerged as key challenges; the allocated time for the sessions was not always sufficient, and scheduling the programme before exams was a distraction for students. “Operation Smoke Storm” faced similar challenges with time. Indeed, the school day is universally a busy day [27]. Teachers employed flexibility to fit the sessions as they saw best, and embedding the SFI into the curriculum was seen as a way to ensure that this important topic is delivered without disrupting the everyday running of the school. Headteachers highlighted some tensions created by delivering an add-on educational activity within a restricted school curriculum and schedule.

During the life course of an individual, early school years play an important role in establishing normative health behaviours, and therefore the primary school offers an important setting. Most teachers and headteachers had been involved in some health promotion activities in the past, and many expressed an interest in partnership working with civil society in advocating and promoting health. It is difficult to say whether this is a general trend in Bangladesh, or if the participating schools and their staff were particularly sensitized to NGO-led health promotion projects. However, this is an encouraging finding for multi-sectorial partnership work in public health in Bangladesh and a useful reminder that early engagement of teachers in the development of school-based health promotion activities is key to capitalise on their enthusiasm, develop effective educational content, and propose meaningful strategies on its delivery.

Informed by this process evaluation and subsequent discussions within the research team, several changes were made to the SFI. The 6-week programme schedule remained, with two 45-min and four 15-min sessions. Despite feedback on time constraints, the didactic components had still been delivered with high fidelity. To enhance good delivery of the interactive components of the SFI, specific student discussions have been built into each session, for example, after the word search, a discussion about SHS facts now follows. Also, the role play has been extended into a drama activity in which children present a play to their parents, designed to educate parents on SHS and engage them in the idea of creating a SFH. The teacher training has been extended from 1 to 2 days to include instruction on behaviour change techniques. Finally, an achievement form has  been designed for the children, where they could tick off tasks relating to their knowledge, confidence and behaviours for example, “I now know how other people’s smoke is harmful for my health”, “I can confidently ask people not to smoke in front of me”.

### Limitations

The key limitation is that we did not formally collect feedback on the SFI from the children and their parents. Instead, headteachers and teachers offered their views on children’s responses to the activities, and we took an objective measure (cotinine levels) to assess the physiological impact of the intervention on children. A cluster randomised controlled trial is now underway, which includes the children’s and parents’ feedback on acceptability and impact.

## Conclusions

Alongside the preliminary evidence of effectiveness and feasibility of study procedures [21], our findings provided useful information to finalise the content and delivery and to inform the scale-up of the SFI for our definitive trial (commenced January 2020).

## Data Availability

The datasets used and/or analysed during the current study are available from the corresponding author on reasonable request.

## Abbreviations

CLASS I: Children Learning About Second-hand Smoke (Feasibility study)
CLASS II: Children Learning About Second-hand Smoke (Pilot cluster randomised controlled trial)
NGO: Non-Governmental Organisation
SFH: Smoke-free homes
SFI: Smoke-free intervention
SHS: Second-hand smoke
